# Hydrophilicity and Pore Structure Enhancement in Polyurethane/Silk Protein–Bismuth Halide Oxide Composite Films for Photocatalytic Degradation of Dye

**DOI:** 10.3390/ijms25126653

**Published:** 2024-06-17

**Authors:** Lingxi Meng, Jian Jian, Dexing Yang, Yixiao Dan, Weijie Sun, Qiuhong Ai, Yusheng Zhang, Hu Zhou

**Affiliations:** 1 School of Chemistry and Chemical Engineering, Hunan Engineering Research Center for Functional Film Materials, Hunan University of Science and Technology, Xiangtan 411201, China; lingximengqr@163.com (L.M.); dyx13536505551@gmail.com (Y.D.); zys_2002@hotmail.com (Y.Z.); 2 School of Chemical Engineering, Xiangtan University, Xiangtan 411105, China; yangdexing2018@163.com (D.Y.); sunweijie2013@163.com (W.S.); aqh168@126.com (Q.A.)

**Keywords:** polyurethane film, silk protein, BiOBr, photocatalytic degradation

## Abstract

Polyurethane/silk protein–bismuth halide oxide composite films were fabricated using a blending-wet phase transformationin situsynthesis method. The crystal structure, micromorphology, and optical properties were conducted using XRD, SEM, and UV-Vis DRS characterize techniques. The results indicated that loaded silk protein enhanced the hydrophilicity and pore structure of the polyurethane composite films. The active species BiOX were observed to grow as nanosheets with high dispersion on the internal skeleton and silk protein surface of the polyurethane–silk protein film. The photocatalytic efficiency of BiOX/PU-SF composite films was assessed through the degradation of Rhodamine B under visible light irradiation. Among the tested films, the BiOBr/PU-SF composite exhibited the highest removal rate of RhB at 98.9%, surpassing the removal rates of 93.7% for the BiOCl/PU-SF composite and 85.6% for the BiOI/PU-SF composite. Furthermore, an active species capture test indicated that superoxide radical (•O_2_^−^) and hole (h^+^) species played a predominant role in the photodegradation process.

## 1. Introduction

In recent years, advancements in science, technology, and the social economy have led to improvements in people’s living standards. However, this progress has also resulted in the emergence of environmental issues, such as the contamination of water bodies with organic pollutants of dye and antibiotics from domestic and industrial sources [1,2]. These pollutants pose a significant threat to both the ecological environment and human health. Rhodamine B (RhB) is recognized as a potentially hazardous dye, as it has been shown to induce a range of health issues such as skin irritation, respiratory tract disorders, and gastrointestinal disorders [3]. Additionally, its presence in aquatic environments has been found to inhibit the photosynthesis process [4]. Therefore, the urgent need to address the removal of RhB dye from aquatic systems has garnered significant attention. 

The utilization of semiconductor photocatalytic technology for the degradation of organic pollutants into CO_2_ and H_2_O has garnered significant interest among researchers due to its cost-effectiveness, energy efficiency, and minimal secondary pollution [5]. Nevertheless, the industrial application potential of traditional photocatalysts such as TiO_2_, ZnO, and other semiconductors isconstrained by their wide band gap and low quantum efficiency [6,7]. Bismuth haloxideBiOX (X = Cl, Br, I) is a ternary compound semiconductor composed of bismuth, oxygen, and halogen atoms. It possesses a distinctive layered structure consisting of [Bi_2_O_2_]^2+^ units interspersed with dihalogen atoms, resulting in weak intermolecular interactions with neighboring halogen atoms. This arrangement leads to an asymmetric charge distribution between layers, creating an internal electrostatic field that promotes the efficient separation of photogenerated electron–hole pairs, thus exhibiting good photocatalytic activity [8,9,10]. However, the photocatalytic performance of the powder nano-granular bismuth halide catalyst is diminished due to the propensity for particle agglomeration during reactions, complicating separation and recovery from the aqueous phase post-reaction and leading to catalyst loss and secondary pollution [11]. Recently, researchers have addressed these challenges by immobilizing powdered nanophotocatalysts onto carriers to create supported nanocomposites, enhancing nanoparticle dispersion and catalyst stability [12]. Zhou et al. [13] achieved a high dispersion of cadmium sulfide nanoparticles on polyurethane films through insitu immobilization, resulting in an enhanced photocatalytic activity and stability for organic pollutant removal. Abad et al. [14] developed an Au/ZnO cellulose film with uniformly dispersed active components, demonstrating a notable 95.28% efficiency in photocatalytic degradation of Eosin Y. 

Polyurethane (PU) is a multi-block polymer known for its superior film-forming characteristics. Polyurethane films exhibit commendable mechanical strength and toughness, utilizing phase conversion technology, rendering them suitable for use as catalyst carriers [15,16]. However, the poor pore structures and hydrophilicity of pure PU film limit its application. Therefore, modifying composite materials with other materials might be an effective way to improve the applicability of PU materials as carriers. Silk fibroin (SF), a naturally derived organic polymer sourced from silkworm cocoons, boasts an abundance of functional groups such as hydroxyl (−OH), amino (−NH_2_), and carboxyl (−COOH). Renowned for its high hydrophilicity, biocompatibility, and tunable mechanical properties, SF has found widespread application in the field of biomedical materials [17,18,19]. Through the modification of pure PU films with SF, the hydrophilicity of the film can be greatly enhanced. Additionally, the substantial disparity in shrinkage rates between polyurethane and silk protein during film formation leads to the creation of larger pore-size film structures, thereby increasing the available loading space for photocatalysts.

Herein, a novel BiOX/PU-SF composite film was successfully prepared using a blending-wet phase conversion methodand in situ precipitation method. The hydrophilicity, micromorphology, phase structure, and optical properties of the composite film were analyzed through various characterization techniques. The photocatalytic performance of the composite films was assessed by simulating the degradation of the pollutant Rhodamine B (RhB) under visible light, and the photocatalytic mechanism was investigated through active species capture experiments and EPR testing.

## 2. Results and Discussion

### 2.1. Structural Characterization

**Figure 1:**
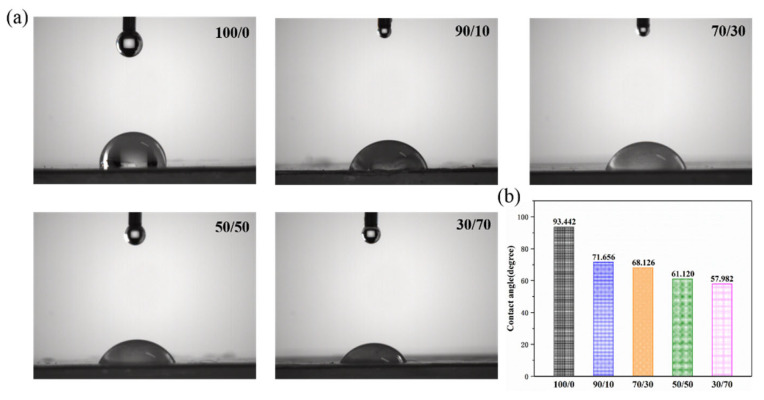
Water contact angle test photographs and values for PU-SF composite films with different proportions.

The hydrophilicity of composite films plays a crucial role in influencing the catalytic activity of photocatalysts [20]. The impact of adding SF to PU on the hydrophilicity of composite films was examined, and the results are illustrated in Figure 1. Clearly, the water contact angle photographs in Figure 1a reveal that the addition of SF significantly improves the hydrophilicity of the PU films. The numerical histogram in Figure 1b shows that the water contact angles of the composite films are 93.442°, 71.656°, 68.126°, 61.120°, 61.120°, and 57.982° when the mass ratio of PU to SF is 100:0, 90:10, 70:30, 50:50, and 30:70, respectively. Obviously, the water contact angle of the PU-SF composite film exhibited a gradual decrease, suggesting an enhancement in hydrophilicity with increasing SF content. The improved hydrophilicity of the composite film through the incorporation of SF can be primarily attributed to the abundance of hydrophilic groups such as −OH, −NH_2_, and −COOH present in the SF particles [17]. This facilitates enhanced contact and reactivity of the active components within the composite film with contaminants present in the aqueous phase. As the proportion of silk fibroin increases to 70%, the hydrophilicity of the PU-SF composite film improves, yet the film-forming efficacy diminishes, leading to a notable decrease in mechanical properties and the occurrence of fragmentation during the reaction process. Consequently, a mass ratio of 50:50 for PU to SF is deemed more appropriate for achieving a PU-SF composite film with optimal hydrophilicity and sustained film-forming capabilities.

**Figure 2:**
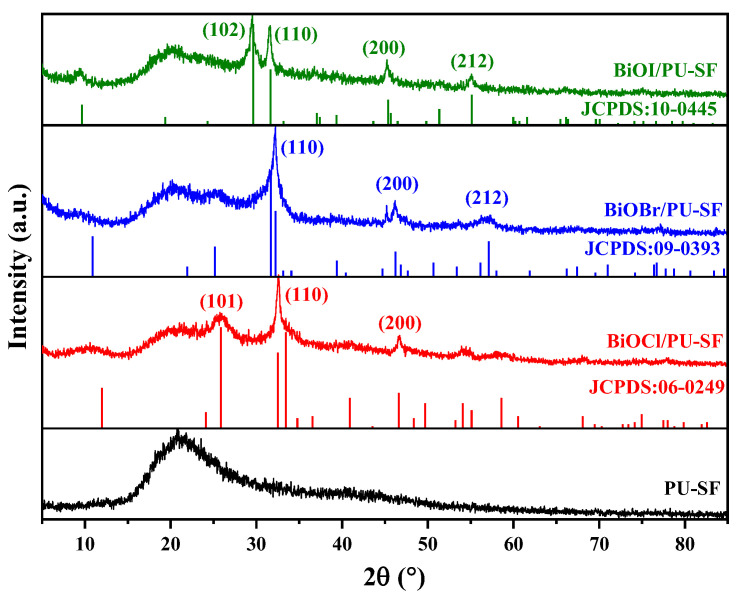
XRD patterns of BiOX/PU-SF (X=Cl, Br, I) composite films.

The composition and crystal structure of PU-SF and BiOX/PU-SF (X=Cl, Br, I) composite films were analyzed, and the results arepresented in Figure 2. In the case of the PU-SF composite film, abroad diffraction peaks at 2θ of 20.5° wereobserved, indicative of the silk II structure of silk proteins [21]. Notably, the diffraction peak corresponding to the silk II structure was reserved in all three samples of BiOX/PU-SF (X=Cl, Br, I), suggesting that the crystal structure of silk proteins remained intact following the in situ precipitation of BiOX particles. Furthermore, the XRD pattern of the BiOCl/PU-SF composite film revealed diffraction peaks at 25.9°, 32.5°, and 46.6°, corresponding to the (101), (110), and (200) crystal faces of the square crystal phase BiOCl (JCPDS No. 06-0249) [22]. Similarly, the diffraction peaks observed in the BiOBr/PU-SF sample at 32.2°, 46.2°, and 57.1° were associated with the (110), (200), and (212) crystal planes of BiOBr (JCPDS No. 09-0393) [23]. The diffraction peaks of the BiOI/PU-SF samples at 29.6°, 31.6°, 45.3°, and 55.1° were in agreement with the (102), (110), (200), and (212) crystal faces of BiOI (JCPDS No. 10-0445) [24]. Moreover, the crystalline sizes of the BiOI/PU-SF, BiOBr/PU-SF, and BiOCl/PU-SF samples calculated using the Scherrer formula were 2.7 nm, 5.1 nm, and 6.5 nm, respectively. The aforementioned results suggested that BiOCl, BiOBr, and BiOI have been effectively incorporated into the PU-SF composite film through an insitu coprecipitation process. The observed sharp peak shapes and absence of heterogeneous peaks indicated the high crystallinity and purity of the active components.

**Figure 3:**
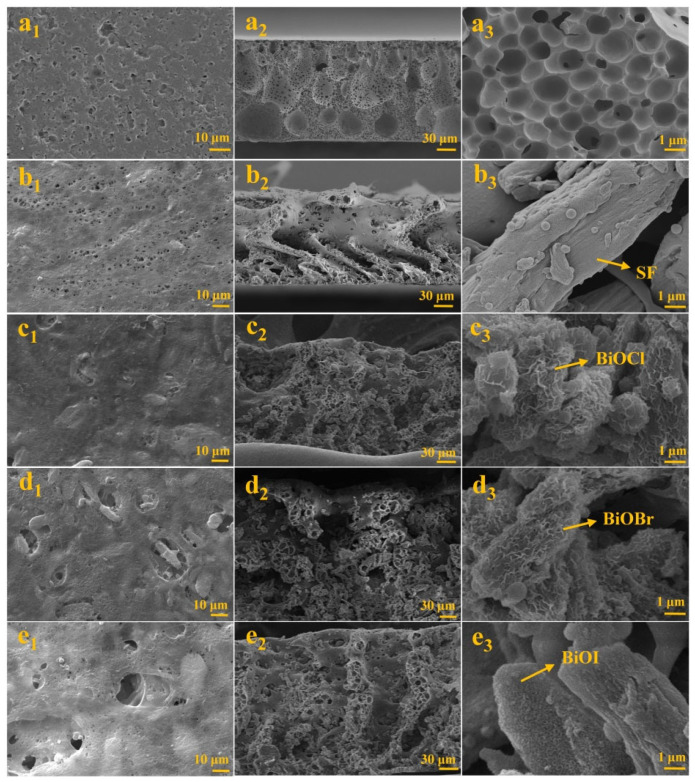
Surface and cross-sectional SEM images of pure PU (**a_1_**–**a_3_**), PU-SF(**b_1_**–**b_3_**), BiOCl/PU-SF(**c_1_**–**c_3_**), BiOBr/PU-SF (**d_1_**–**d_3_**), BiOI/PU-SF (**e_1_**–**e_3_**) composite films.

Figure 3 displays the surface and cross-sectional SEM images of the pure PU, PU- SF, and BiOX/PU-SF (X=Cl, Br, I) composite films. The surface of the pure PU film exhibits minimal pore structures and limited internal connectivity, as depicted in Figure 3(a_1_–a_3_). In contrast, the PU-SF surface displays pore structures with a diameter of approximately 1–3 μm and well-established internal connectivity, as shown in Figure 3(b_1_–b_3_). The cross-sectional SEM image of pure PU reveals a honeycomb-like pore structure enclosed by the film skeleton in its internal morphology, and the thickness of the film was measured to be 180 μm. Following the modification with silk fibroin, the PU-SF composite film exhibits the emergence of a substantial cavity structure resembling a large finger hole, along with enhanced connectivity between pores and between the interior and exterior of the film. Furthermore, the distribution of SF particles is observed in the internal framework of the film. This phenomenon can be attributed to the disruptive effect of silk protein particles on the continuous phase of polyurethane, resulting in the destabilization of the polyurethane solution. Consequently, during the film-forming process, the presence of SF promotes phase separation between SF and PU, ultimately leading to the formation of larger, more porous structures with improved connectivity. This not only increases the loading capacity for active species but also enhances the diffusion rate of pollutants in the aqueous phase within the film and improves contact efficiency with photocatalysts [25,26]. When various types of BiOX (X=Cl, Br, I) are incorporated into the film, the internal cavities of the film become filled, resulting in the formation of nanosheet-like structures of varying sizes on the surface of SF, as shown in Figure 3(c_2_,c_3_,d_2_,d_3_,e_2_,e_3_). Among them, the particle size and structure of BiOCl and BiOBr nanosheets in BiOCl/PU-SF and BiOBr/PU-SF composite films exhibit significant similarities, whereas BiOI in BiOI/PU-SF is present in the film skeleton and SF surface as denser small flake particles, resembling the morphology of BiOX as reported in existing literature [27]. It is noteworthy that the active component BiOX is well dispersed within the films without noticeable agglomeration, suggesting that incorporating nanostructured BiOX into the porous PU-SF composite film effectively mitigates the issue of solid particle agglomeration.

**Figure 4:**
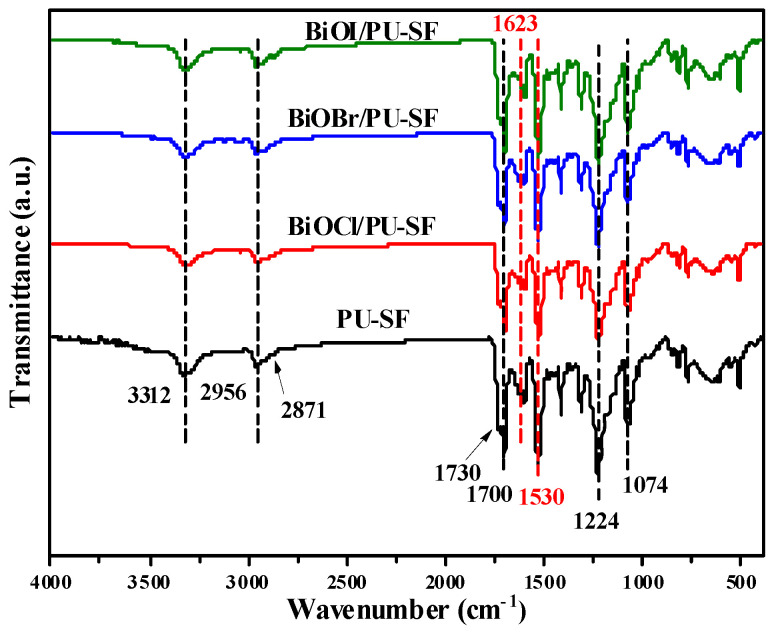
FT-IR spectra of PU-SF and BiOX/PU-SF (X=Cl, Br, I) composite films.

Figure 4 displays the FT-IR spectra of PU-SF and various BiOX/PU-SF (X=Cl, Br, I) composite films. The PU-SF film reveals an absorption peak at 3312 cm^−1^ corresponding to the N-H stretching vibration in PU, along with the C-H stretching vibration at 2956 cm^−1^, the -CH_2_ symmetric stretching vibration at 2871 cm^−1^, and characteristic peaks at 1730 cm^−1^ and 1700 cm^−1^, representing the free C=O stretching vibration and hydrogen bond-associated carbonyl stretching vibration, respectively [28]. The band observed at approximately 1530 cm^−1^ is attributed to the overlapping of the -NHCO adsorption peak of PU with the amide II peak of silk protein. Both the peaks at 1224 cm^−1^ and 1074 cm^−1^ are identified as C-O-C telescopic vibrations [25]. Furthermore, a distinctive band corresponding to amide I is evident at 1623 cm^−1^, indicative of the anti-parallel β-folded structure of silk II [29]. The absorption peaks of the composite film remained largely unchanged following the incorporation of BiOX, potentially attributable to the masking effect of the Bi-O vibration characteristic peak within the 500–530 cm^−1^ region. This minimal alteration indicates that the in situ loading of BiOX (X=Cl, Br, I) does not significantly alter the structure of the PU-SF composite film, which is consistent with XRD characterization results.

**Figure 5:**
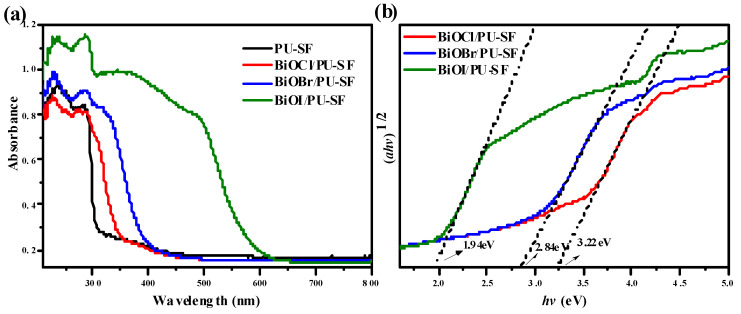
UV-vis DRS spectra (**a**) and (*αhν*)^1/2^ ~*hν* curve (**b**) of BiOX/PU-SF (X=Cl, Br, I) composite films.

Figure 5 shows the UV-vis DRS spectra of PU-SF and different types of BiOX/PU-SF (X=Cl, Br, I) composite films. Figure 5a illustrates that the PU-SF composite film predominantly absorbs in the ultraviolet region, with an absorption edge at approximately 310 nm, primarily due to the presence of aromatic amino acids such as tryptophan, tyrosine, and phenylalanine in silk protein [30]. In comparison, the BiOCl/PU-SF, BiOBr/PU-SF, and BiOI/PU-SF composite films exhibit significantly enhanced light absorption capabilities, displaying absorption of ultraviolet light, weak visible light, and strong visible light, with absorption edges at approximately 356 nm, 410 nm, and 615 nm, respectively. The bandgap width of semiconductor materials can be determined using the Tauc formula [31,32] *αhν* = A(*hν*-*E*_g_)^n^, where *α*, *h*, *ν*, A, and *E*_g_ denote the absorption coefficient, Planck constant, absorption spectral rate, scale factor, and bandgap of the semiconductor, respectively. The n values are uniformly assigned as 2 because the BiOCl, BiOBr, and BiOI are the indirect transition bandgap semiconductors [33]. Utilizing this formula, the band gap values of (*αhν*)^1/2^~*hν* band gap calculation chart (Figure 5b) were calculated, resulting in band gap values of 3.22 eV, 2.84 eV, and 1.94 eV for BiOCl/PU-SF, BiOBr/PU-SF, and BiOI/PU-SF composite films, respectively, which are consistent with the literature values [34].

### 2.2. Photocatalytic Efficiency

**Figure 6:**
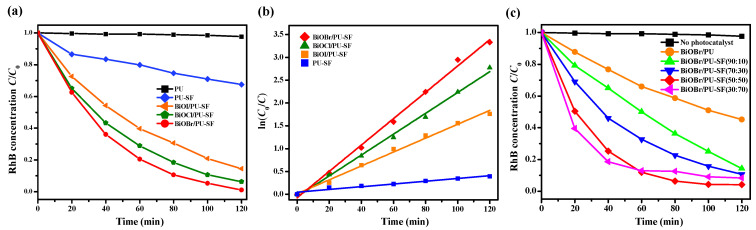
Photocatalytic performance of pure PU, PU-SF, BiOX/PU-SF (X=Cl, Br, I) composite films (**a**); related degradation rate plots (**b**); photocatalytic performance with different PU and SF proportions of BiOBr/PU-SF composite films (**c**).

The photocatalytic performances of BiOX/PU-SF (X=Cl, Br, I) composite films were assessed through the degradation of RhB under visible light irradiation; the results are presented in Figure 6. Figure 6a revealed that the concentration of RhB remained relatively constant when pure PU film was used as the catalyst, indicating minimal photocatalytic activity in visible light. In contrast, when the PU-SF composite film was employed as the catalyst, the RhB concentration decreased to 32.5% after 120 min, potentially due to the strong adsorption capabilities of the PU-SF composite film on RhB. The degradation efficiency of BiOX/PU-SF (X=Cl, Br, I) composite films was significantly enhanced, with BiOBr/PU-SF composite film exhibiting the highest degradation rate of 98.9 ± 1.0% for RhB. This was followed by BiOCl film with a degradation rate of 93.7 ± 2.5%, while the BiOI composite film showed the lowest activity at 85.6 ± 2.0%. It can be seen from the characterization of UV-vis DRS that the BiOCl composite film exhibits strong ultraviolet light absorption but demonstrated effective photocatalytic activity against RhB under visible light conditions, primarily due to the indirect dye photosensitization process in the photocatalytic reaction. While BiOBr/PU-SF presents limited visible light-absorption capability, the visible light catalytic degradation of RhB may be attributed to both direct photocatalytic degradation and indirect dye photosensitization processes, leading to greater photocatalytic activity compared to BiOCl/PU-SF [35]. In contrast, despite the enhanced visible light-absorption ability of BiOI/PU-SF composite films, the conduction band potential of BiOI is excessively positive and the valence band potential is excessively negative. This valence band structure diminishes its oxidation-reduction capacity, resulting in the lowest photocatalytic activity [36,37]. Furthermore, the degradation kinetics of RhB were investigated utilizing the pseudo-first-order kinetic model (In(*C*_0_/*C*) = *k*t) [38]. In Figure 6b, the kinetic fitting correlation coefficient R^2^ of all composite films exceeded 0.99, indicating that the degradation of RhB follows the first-order kinetic model. Additionally, the reaction rate constants of BiOCl/PU-SF, BiOBr/PU-SF, and BiOI/PU-SF composite films were determined to be 0.0228, 0.0289, and 0.0152 min^−1^, respectively. These values were found to be 7.57, 9.60, and 5.05 times higher than those of PU-SF composite films, respectively.

Moreover, the impact of the hydrophilicity and pore structure of the composite films on the photocatalytic efficacy of the catalysts was examined through variations in the mass ratio of PU and SF; the results areshown in Figure 6c. It is evident that the concentration of RhB remains relatively constant in the absence of a catalyst, suggesting minimal self-degradation of RhB under visible light exposure. The degradation rate of RhB in BiOBr/PU composite films prepared by directly loading BiOBr into pure PU films was only 54.8%, while the photocatalytic activity of the composite films after SF modification was significantly improved. Specifically, the degradation rates of RhB in BiOBr/PU-SF (90:10), BiOBr/PU-SF (70:30), and BiOBr/PU-SF (50:50) composite films were 85.7%, 89.3%, and 98.9%, respectively. This can be attributed to the inherent characteristics of pure PU film, such as limited pore structures, low connectivity, and reduced hydrophilicity, which hinder the diffusion of RhB within the film and limit its contact efficiency with BiOBr. The introduction of SF not only enhances the hydrophilicity of the composite film but also facilitates the formation of a well-connected pore structure that promotes the flow of pollutants within the film and improves the contact efficiency with the active component BiOBr. In addition, it has been reported that SF particles exhibit a high adsorption capacity for RhB, thereby enhancing the contact efficiency and adsorption performance of catalysts and pollutants [39]. Nevertheless, as the SF ratio increased to 70%, the degradation rate of the BiOBr/PU-SF (30:70) composite film for RhB decreased to 91.6%. This phenomenon can be attributed to the increased phase separation at higher SF proportions, resulting in reduced incorporation of SF particles within the PU backbone. Consequently, SF particles detached to some extent, leading to film fragmentation during the reaction process and impacting the photocatalytic degradation process.

**Figure 7:**
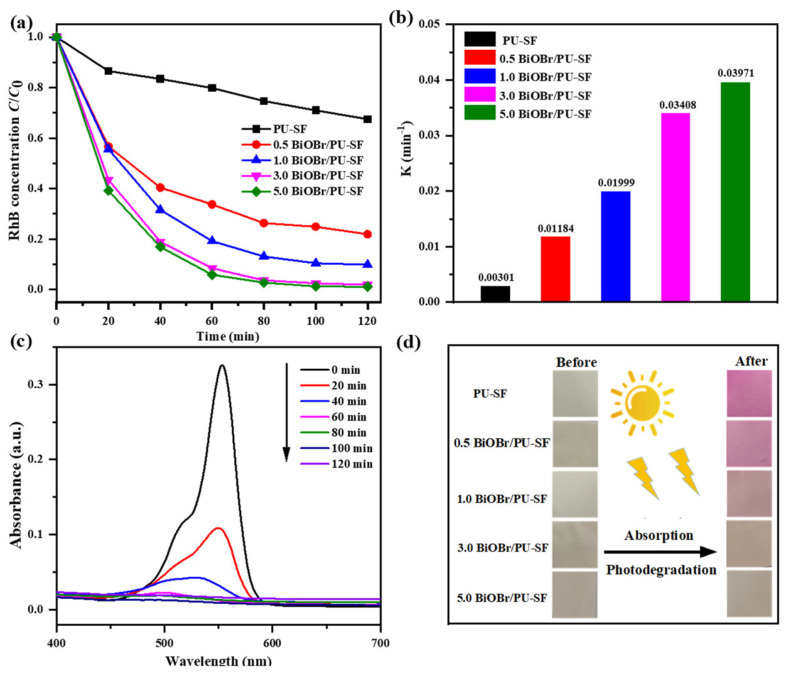
Photocatalytic performance of BiOBr/PU-SF composite films with different BiOBr loading (**a**); first-order dynamic linear fitting K-value (**b**); absorption spectra changes of RhB solution under visible light irradiation over 5.0BiOBr/PU-SF catalyst (**c**); color changes of composite films before and after reaction (**d**).

Figure 7a shows the effect of different BiOBr loadings on the photocatalytic performance of BiOBr/PU-SF composite films. The enhancement of photocatalytic activity in PU/SF BiOBr composite films is directly correlated with the loading amount of BiOBr. Specifically, the degradation rates of RhB increase from 78.1% to 98.9% as the loading amount of BiOBr is raised from 0.5 mmol to 5.0 mmol. However, beyond a certain threshold, further increases in loading amount do not significantly impact the photocatalytic activity. This phenomenon may be attributed to the saturation of pores within the PU-SF film as the loading amount of BiOBr increases, rendering additional loading content ineffective in enhancing photocatalytic performance. Furthermore, the degradation kinetics constants of different BiOBr loading levels are 0.01184, 0.01999, 0.03408, and 0.03971 min^−1^ (in Figure 7b), which are 3.92, 6.64, 11.33, and 13.19 times higher than those of pure PU-SF composite film, respectively. This indicates that the photocatalytic activity significantly increases with the increase inBiOBr loading levels.Figure 7c illustrates the dynamic absorption spectrum of the 5.0BiOBr/PU-SF composite film in RhB solution under visible light irradiation. The absorbance of the RhB solution at the maximum absorption wavelength of 555 nm shows a notable decrease over time, ultimately reaching complete disappearance after 80 min. This suggests effective degradation of RhB within the solution, without the formation of any new derivatives throughout the photocatalytic process. Moreover, a comparison was made between the color changes of the composite film before and following utilization in the photocatalytic process, as illustrated in Figure 7d. It is evident that the PU-SF composite film exhibited the most pronounced staining by RhB as a result of its exclusive adsorption effect. With the incremental increase in BiOBr loading, the observable color change of the composite film diminished before and after application. This suggests that the RhB dye undergoes mineralization rather than mere adsorption on the composite films. Furthermore, the mineralization efficiency of the RhB solution was confirmed through total organic carbon (TOC) analysis using the 5.0BiOBr/PU-SF composite film photocatalyst. The result demonstrated a TOC removal rate of approximately 95.72%, indicating the catalytic degradation of RhB dye into CO_2_ and H_2_O, as well as the presence of intermediate RhB molecules in the medium.

**Figure 8:**
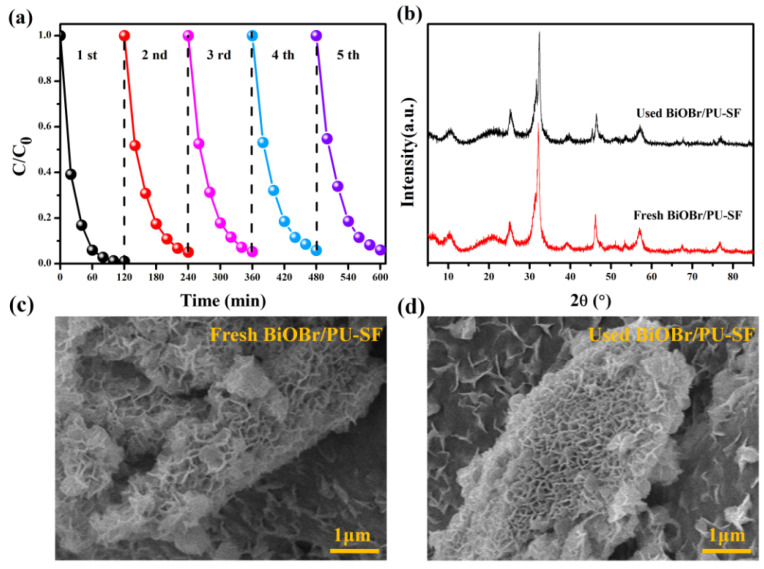
Cycling runs of photocatalytic degradation of RhB in the presence of BiOBr/PU-SF composite film (**a**), and the XRD patterns (**b**) and cross-sectional SEM images of fresh and used BiOBr/PU-SF composite film (**c**,**d**).

The importance of catalyst stability and reusability in practical applications necessitated an investigation into the cyclic experiment of BiOBr/PU-SF composite film photocatalytic degradation of RhB. Figure 8a reveals a gradual decrease in the degradation rate of the composite film from 98.9% in the initial cycle to 94.0% in the fifth cycle, with subsequent stabilization as the number of cycles increased. The results suggest that the BiOBr/PU-SF composite film exhibits sustained stability during the visible light degradation of RhB. In addition, the XRD spectra and SEM images of the BiOBr/PU-SF composite film in Figure 8b–dreveal that the crystal phase structure and morphology of the catalyst remain consistent before and after recycling. These resultsindicate that the BiOBr/PU-SF composite film exhibits stability in the photocatalytic degradation of RhB. At the same time, the composite film is easy to separate and recover from the aqueous phase without the need for centrifugation or filtration, thereby effectively mitigating the risk of secondary pollution resulting from nanoparticle loss.

### 2.3. Possible Photocatalytic Mechanism

**Figure 9:**
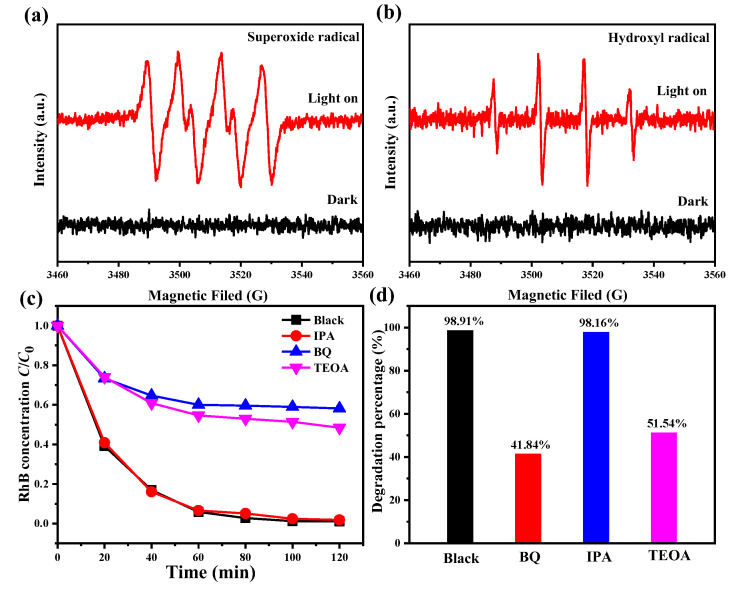
EPR spectra of DMPO-•O_2_^−^(**a**) and DMPO-•OH (**b**) containing BiOBr/PU-SF catalyst, and active species trapping experiments of RhB photodegradation over BiOBr/PU-SF(**c**,**d**).

The superoxide radicals (•O_2_^−^) and hydroxyl radicals (•OH) in the photocatalytic reaction system of BiOBr/PU-SF composite film were detected using electron spin resonance (EPR) technology, with DMPO as the capturing agent. In Figure 9a,b, the presence of four characteristic peaks of •OH and six characteristic peaks of •O_2_^−^ can be observed during visible light irradiation, while no distinct peaks are evident under conditions of no light [40]. This suggests that the BiOBr/PU-SF composite film is capable of generating •OH and •O_2_^−^ effectively during the photocatalytic process. Moreover, the active species generated during the catalytic degradation of RhB by the BiOBr/PU-SF composite film were identified through an active species capture experiment. P-benzoquinone (BQ), isopropanol (IPA), and triethanolamine (TEOA) were used to capture superoxide radicals (•O_2_^−^), hydroxyl radicals (•OH), and holes (h^+^), respectively. As shown in Figure 9c,d,the addition of IPA did not significantly impact the degradation efficiency of RhB, suggesting that hydroxyl radicals (•OH) were not the primary active species responsible for the degradation of RhB by the BiOBr/PU-SF composite film. Nevertheless, the presence of TEOA and BQ resulted in a decrease in the degradation rate of RhB by the BiOBr/PU-SF composite film from 98.9% to 51.5% and 41.8%, respectively. This inhibition of the degradation process suggests that •O_2_^−^ and h^+^ are the primary active species involved. The combination of these experimental results indicates that BiOBr/PU-SF composite films can generate •OH and •O_2_^−^ during the photocatalytic degradation of RhB, but •O_2_^−^ and h^+^ play a predominant role in the photocatalytic reactions.

**Scheme 1:**
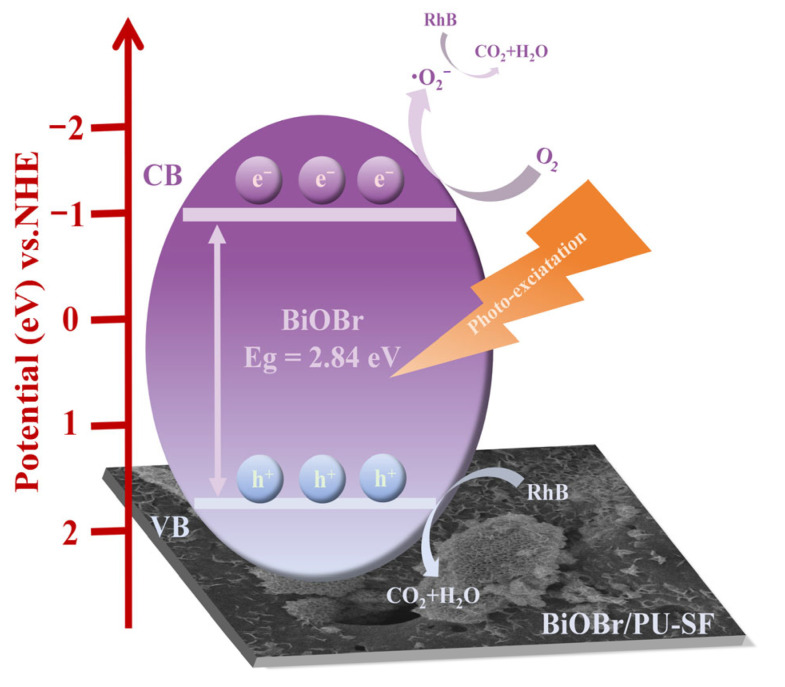
Proposed mechanism for RhB photodegradation under visible light irradiation over a BiOBr/PU-SF composite film.

Based on the aforementioned results, a potential photocatalytic reaction mechanism for the degradation of RhB using a BiOBr/PU-SF composite film was proposed, as illustrated in Scheme 1. The bandgap width of BiOBrwas determined to be 2.84 eV through the Kubelka Munk formula and UV-vis DRS spectra, and valence band (VB) and conduction band (CB) edge potentials were calculated using empirical formulas [41,42] *E*_VB_ = *X* − *E*_e_+ 0.5*E*_g_ and *E*_CB_ = *E*_VB_ − *E*_g_, where *E*_VB_ and *E*_CB_ are the VB and CB edge potentials, respectively, and *X* denotes the absolute electronegativity of BiOBr at 4.885 eV [21]. *E*_e_ is the energy of the free electron versus hydrogen at 4.5 eV, and *E*_g_ is the bandgap width of 2.84 eV. Therefore, the *E*_VB_ and *E*_CB_ values were approximately 1.805 eV and −1.035 eV (vs. NHE), respectively. Upon irradiation with visible light, the BiOBr/PU-SF composite film activates the semiconductor BiOBr, resulting in the generation of electron–hole pairs. The excited electrons in the BiOBr valence band transition to the conduction band, while the remaining h^+^ in the valence band undergoes a redox reaction with RhB to produce CO_2_ and H_2_O. The CB_BiOBr_ (−1.035 eV) is lower than the standard potential of O_2_/•O_2_^−^ (−0.33 eV vs. NHE) [43], facilitating the conversion of O_2_ molecules to •O_2_^−^ and enabling the efficient oxidation of RhB. However, the VB_BiOBr_ potential (1.805 eV) is lower than the H_2_O/•OH potential (2.4 V) [44]; the holes in the VB of BiOBr cannot oxidize H_2_O to •OH, resulting in limited •OH production during degradation.

## 3. Experimental

### 3.1. Materials

PU was provided by the National Engineering Laboratory for Clean Technology of Leather Manufacture at Sichuan University, Chengdu, China. SF was obtained from the Sinopharm Chemical Reagent Co. Ltd., Shanghai, China. *N*,*N*-dimethylformamide (DMF), bismuth nitrate pentahydrate (Bi(NO_3_)_3_·5H_2_O), ammonium bromide (NH_4_Br), ammonium chloride (NH_4_Cl), potassium iodide (KI), and glacial acetic acid (C_2_H_4_O_2_) were purchased from Aladdin Biochemical Technology Co. Ltd., Shanghai, China. Benzoquinone (BQ), triethanolamine (TEOA), isopropanol (IPA), and rhodamine B (RhB) were supplied by Xilong Scientific Co. Ltd., Shantou, China. Distilled water was self-made in the laboratory.

### 3.2. In Situ Synthesis of BiOX/PU-SF Composite Films

**Scheme 2:**
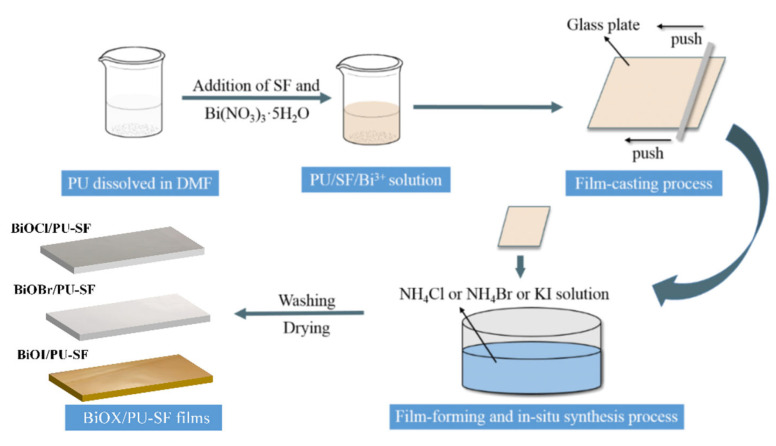
Schematic diagram for the preparation process of BiOX/PU-SF composite films.

BiOX/PU-SF composite films were prepared by blending-wet phase transformation and in situ precipitation method. The schematic of the preparation process isdisplayed in Scheme 2. Typically, 2.5 g of PU and 2.5 g of SF were completely dissolved in 20.0 g of DMF by constant stirring at room temperature. Then, 0.97 g (2 mmol) of Bi(NO_3_)_3_·5H_2_O was added into the above solution and stirred for 2 h. The obtained solution was subjected to ultrasound for 10 min to remove bubbles. Simultaneously, NH_4_Br, NH_4_Cl, and KI aqueous solutions with the same molar ratio of Bi and X (Br, Cl, and I) were prepared, respectively, and the pH was adjusted to 2 using glacial acetic acid. After that, the above-deaerated solutions were coated onto the glass plate and rapidly transferred to the as-prepared NH_4_Br, NH_4_Cl, or KI solution at room temperature for 6 h to in situ synthesized BiOBr, BiOCl, or BiOI. Subsequently, the obtained films were washed with distilled water several times and dried in a vacuum oven at 40 °C for 12 h. Finally, the obtained composite films were labeled as BiOBr/PU-SF, BiOCl/PU-SF, and BiOI/PU-SF, respectively. In addition, the different BiOBr loadings in the composite films are determined based on the molar amounts of Bi(NO_3_)_3_·5H_2_O and NH_4_Br added.

### 3.3. Characterization

The XRD characterization was analyzed by theRigakuD/Max 2550 VB+18 KW X-ray diffractometer using Cu-Kα irradiation with a working current of 40 mA and a voltage of 30 kV. The morphology was observed using an SEM system (Zeiss Sigma 300 type, Oberkochen, Germany). The FT-IR spectroscopy was conducted on a Germany Bruker TENSOR II. The UV-vis DRS spectra were recorded on a Japan Shimadzu UV-2550. The contact angle was tested on a JC200D1 contact angle measuring instrument. The electron paramagnetic resonance spectrometer (EPR) was the Bruker EMXplus EPR spectrometer.

### 3.4. Evaluation of Photocatalytic Activity

The photocatalytic performances of BiOX/PU-SF composite films was evaluated by the degradation of RhB under visible light irradiationusing a 300 W Pofelet xenon lamp light source equipped with a UV cutoff filter, and the wavelength range was 420–780 nm. The light intensity measured by a radiometer was 25.5 mW/cm^2^. Generally, 0.2 g of composite film was added to 200 mL of RhB solution (10 mg/L). Prior to irradiation, the suspensions were left for 30 min in the dark to achieve equilibrium for adsorption–desorption. After turning on the lamp, 10 mL of the reaction solution was sampled every 20 min and centrifuged to remove any nanoparticles that may have peeled off from the composite film. Subsequently, the residual concentrations of RhBwereanalyzed immediately using a UV mini-1240 spectrophotometer at the corresponding maximum absorption wavelength. Moreover, the total organic carbon (TOC) was measured using a TOC-L analyzer (Shimadzu, Japan). After the reaction, the composite film was directly taken out, washed with distilled water several times, and dried at 40 °C for 12 h, and then the next run was tested to investigate the catalyst stability. 

## 4. Conclusions

A set of BiOX/PU-SF (X=Cl, Br, I) composite films was effectively fabricated through a simultaneous wet phase transformation and in situ synthesis approach. The characterization results demonstrate that the incorporation of SF enhances the pore architecture of the PU film, creating ample room for the in situ incorporation of the active BiOX species. Moreover, the presence of numerous hydrophilic groups in SF significantly improves the hydrophilicity of the PU film and enhances the connectivity between its interior and exterior. Furthermore, the degradation efficiency of RhB decreases in the following order: BiOBr/PU-SF > BiOCl/PU-SF > BiOI/PU-SF; this is attributed to the light absorption properties of BiOX, the indirect dye photosensitization process, and the conduction band valence band position. A 98.9% removal rate of RhB was achieved under visible light irradiation with BiOBr/PU-SF composite film as the catalyst, and a degradation rate of over 94% was maintained after five cycles of reuse. The composite film exhibits high stability in photocatalytic activity and can be conveniently recovered from wastewater solutions for recycling and reuse, indicating promising practical applications.

## Data Availability

Data are contained within the article.
